# Supplementary material for: Advancing Transgender Health Education for Health Professional Students: A Faculty Fellowship Model for Capacity Building and Curricular Change
Source: Healthcare (Basel). 2025 Aug 26;13(17):2124. doi: 10.3390/healthcare13172124 (PMC12427805; doi:10.3390/healthcare13172124)
Supplement: Supplementary file 1 [file healthcare-13-02124-s001.zip › healthcare-3753133-supplementary.pdf]

Supplement File S1. Curricular Map of Transgender Health Content Throughout Seven Health Professions.

Content	Physician Assistant	Nursing	Athletic Training	Nutrition and Dietetics	Clinical Psychology	Marriage & Family Therapy	Social Work
Gender identity development	**Course**: Essentials of Pediatrics; Clinical Pediatrics<br><br>**Content**: Development of gender identity through childhood and adolescence<br><br>**LO**: Define key terms related to gender identity.<br><br>Describe the development of gender identity through childhood.<br><br>**Reading:** Gender identity development in children<br><br>Development of gender identity<br><br>**Activity:** Guest speaker - clinician who works in gender-affirming healthcare describing their role and available medical services for gender-diverse children and adults	**Course:** Human Growth and Development across the Lifespan<br><br>**Content:** Gender identity development during childhood through adolescence<br><br>**LO:** Describe the development of gender identity during childhood, including familial, cultural, and societal influences.<br><br>**Reading:** Gender Galaxy from Action Canada<br><br>**Activity:** Small group break-out session; discussion and reflection on personal gendered or non-gendered childhood experiences	**Course:** Human Growth and Development<br><br>**Content:** Gender identity development throughout the lifespan<br><br>**LO:** Describe the development of gender identity through different life stages.<br><br>Define terms used to appropriately describe gender diversity and expansive expressions.<br><br>Discuss the role of gender identity in group (family, peer, team sport) socialization.<br><br>**Reading:** Gender Galaxy from Action Canada<br><br>**Activity:** Small group reflection and discussion	**Course**: Nutrition in the Life Cycle<br><br>**Content**: Gender identity development and implications for eating patterns in different life stages<br><br>**LO**: Identify the influences on eating patterns at various life stages, including gender.<br><br>**Reading:** Assessing gender differences in food preferences and physical activity: a population-based survey<br><br>**Activity:** Small group reflection and discussion	**Courses**: Lifespan Development; Human Diversity; Psychopathology<br><br>**Content**: Gender identity development as part of human development; history of pathologization of gender minorities<br><br>**LO**: Discuss gender identity development in context of human development<br><br>**Reading**: Gender identity development among transgender and gender nonconforming emerging adults: An intersectional approach<br><br>Patterns of Gender Development<br><br>Early Childhood Development: A Multicultural Perspective, 8th Edition<br><br>**Activity**: Group discussion on gender identity development; incorporate TGD experiences in course papers	**Course:** Human Development and Lifespan<br><br>**Content:** Gender identity development within human development, inclusive of family gender socialization and cultural practices<br><br>**LO:** Describe the development of gender identity during childhood, including familial, cultural, and societal influences.<br><br>**Reading:** Gender identity in childhood: A review of the literature<br><br>Key aspects in the development of gender identity and sexual orientation according to trans and gender diverse people: a qualitative approach<br><br>Gender Galaxy from Action Canada<br><br>**Activity:** Small group reflection and discussion	**Course**: Human Behavior and Social Environment<br><br>**Content**: Gender identity development inclusive of gender diversity in a heteronormative social environment, family gender socialization, and cultural practices.<br><br>**LO**: Engage diversity and difference in social work practice.<br><br>Assess, intervene, and evaluate practice with individuals, families, groups, organizations, and communities with an LGBTQ+ affirming approach.<br><br>**Reading**: Transgender Theory for Contemporary Social Work Practice: A Question of Values and Ethics<br><br>Bending Gender, Ending Gender: Theoretical Foundations for Social Work Practice with the Transgender Community<br><br>Harnessing the Lived Experience of Transgender and Gender Diverse People as Practice Knowledge in Social Work: A Standpoint Analysis - Hannah Kia, Kinnon Ross MacKinnon, Kaan Göncü, 2023;<br><br>HRC | Understanding the Transgender Community<br><br>**Activity**: Small group reflection and discussion

	Physician Assistant	Nursing	Athletic Training	Nutrition and Dietetics	Clinical Psychology	Marriage & Family Therapy	Social Work
Gender minority populations	**Course**: Professional and Psychosocial Issues in PA Practice<br><br>**Content**: Overview of LGBTQ+ populations, demographics, and health risks<br><br>**LO:** Discuss the experiences and attitudes of members of the LGBTQ community in the United States.<br><br>Describe challenges clinicians may face in caring for members of the LGBTQ+ community<br><br>Explain communication techniques that will enhance the provider-patient relationship when caring for gender minority populations.<br><br>**Reading:** The State of the LGBTQ Community in 2020<br><br>Cultural Competence in the Care of LGBTQ Patients<br><br>**Activity:** Critical reflection paper discussing the author's observed experiences in healthcare and their recommendations to improve such care.	**Course:** Human Growth and Development across the Lifespan<br><br>**Content:** Introduction to gender diverse and LGBTQ+ terminology, demographics, health risks, lifespan barriers<br><br>**LO:** Define the correct terminology with regard to gender diversity<br>Describe gender-affirmative health care.<br><br>Describe the health disparities/barriers to healthy development TGD persons may face.<br><br>**Reading:** Trans-Affirming Language Guide<br><br>**Activity:** Small group break-out session on first awareness of TGD persons; introductory case study on gender-affirming introductions in the health care setting	**Course:** Healthcare Ethics<br><br>**Content:** Ethical considerations and best practices for patient care of gender minority populations<br><br>**LO:** Discuss the impact of gender-inclusive care on health outcomes of gender minority populations.<br><br>Describe gender-inclusive patient care best practices<br><br>**Reading**: The Role of the Athletic Trainer in Providing Care to Transgender and Gender-Diverse Patients: Foundational Knowledge and Disparities-Part I<br><br>**Activity:** Critical reflection of a future role as an athletic trainer in gender-affirming care and support for gender identity development.	**Course:** Community Nutrition<br><br>**Content:** Overview of LGBTQ+ populations, demographics, and nutrition and health risks<br><br>**LO:** Identify nutrition-related psychosocial considerations for transgender people living in the United States.<br><br>**Reading:** Caring for Transgender Patients and Clients: Nutrition-Related Clinical and Psychosocial Considerations<br><br>**Activity:** Design a PSA that addresses a nutrition concern for the transgender community.	**Course**: Clinical Vertical Teams (CVT); Clinical Interventions; Professional Development Seminar; Human Diversity<br><br>**Content**: Best practices for working with TGNC individuals; TGD considerations for treatment<br><br>**LO**: Identify at least 3 barriers TGD individuals may encounter and ways to provide support for these<br><br>Identify at least 3 considerations/ modifications that may be needed for TGD individuals across different treatment modalities (e.g., CBT, IPT, etc.)<br><br>**Readings**:<br>Guidelines for Psychological Practice With Transgender and Gender Nonconforming People<br><br>Advancing the Practice of Pediatric Psychology with Transgender Youth: State of the Science, Ongoing Controversies, and Future Directions<br><br>Identity as Resistance: Identity Formation at the Intersection of Race, Gender Identity, and Sexual Orientation<br><br>**Activity**: Create case conceptualization of presenting concerns and treatment considerations for TGNC individuals	**Course:** Foundations of Multicultural Family Therapy<br><br>**Content:** TGD people and their families; language and terminology<br><br>**LO:** Describe LGBTQ+ communities and their families.<br><br>**Reading:**<br>Understanding Transgender and Non-binary Youth Mental Health Through the Family Resilience Framework: A Literature Review.<br><br>LGBTQ Parent Families<br><br>**Activity:** Watch video interviews with TGD parents; complete reflections and application to alliance building with LGBTQ parents and families.	**Course**: Values and Ethics; Anti-Racism in Social Work Practice<br><br>**Content**: Demonstrate ethical and professional behavior consistent with the social work values and ethics in specialized areas of practice.<br><br>**LO**: Make ethical decisions using the standards of the NASW Code of Ethics, relevant laws and regulations, models for ethical decision-making, ethical conduct of research, and additional codes of ethics as appropriate to the context in specialized areas of practice.<br><br>**Readings**:<br>NASW-Institute Ethical Considerations with LCGBTQ+ Clients<br><br>Affirmative Social Work and Supervision for Transgender and Non-binary Clients<br><br>**Activity**: Case study of a transgender client that applies models for ethical decision-making

	Physician Assistant	Nursing	Athletic Training	Nutrition and Dietetics	Clinical Psychology	Marriage & Family Therapy	Social Work
Historical and social context of TGD people	**Course**: Professional and Psychosocial Issues in PA Practice<br><br>**Content**: Recognizing history of LGBTQ+ individuals in the United States with an emphasis on discrimination in the healthcare system and its impact on health outcomes<br><br>**LO:** Describe the history of transgender individuals with an emphasis on transgender healthcare.<br><br>**Reading:** How Historians are Documenting the Lives of Transgender People<br><br>The Evolution of Transgender Health Care<br><br>**Activity:** Watch a documentary that tells the story of a transgender individual, followed by small group discussions of the movie and the broader context of the TGD experience in the United States	**Course:** Health Promotion; Public Health Nursing<br><br>**Content:** Historical/social context of LGBTQ+ populations with an emphasis on stigma, bias, and discrimination in relation to health outcomes<br><br>**LO:** Identify barriers to healthcare and health risks for LGBTQ+ populations.<br><br>Discuss the lived experience and health care needs of the LGBTQ+ population.<br><br>**Reading:** Considerations for the Care of Transgender Individuals<br><br>Room Assignments, Gender Identity, and Gender Expression: A Case Study on Caring for Transgender Patients<br><br>**Activity:** Individual reflection on assigned readings	**Course:** Sport Psychology<br><br>**Content:** Historical and social context of TGD people, particularly in healthcare; health disparities of TGD people.<br><br>**LO:** Discuss the role healthcare has played in the historical/social trajectory of TGD people.<br><br>Describe historical and existing health disparities of TGD people.<br><br>Discuss the role of gender identity in group (family, peer, sport) socialization.<br><br>**Reading:** Sport and Transgender People: A Systematic Review of the Literature Relating to Sport Participation and Competitive Sport Policies<br><br>**Activity:** Simulated patient interactions performed in front of class with integrated feedback; simulated patients represent diverse backgrounds with psychological issues commonly seen in athletic training practice	**Course:** Healthcare Ethics<br><br>**Content:** Historical and social context of TGD people, particularly in healthcare; health disparities of TGD people.<br><br>**LO:** Discuss the role healthcare has played in the historical/social trajectory of TGD people.<br><br>**Reading:** How Historians are Documenting the Lives of Transgender People<br><br>The Evolution of Transgender Health Care<br><br>**Activity:** Watch a documentary that tells the story of a transgender individual, followed by small group discussions of the movie and the broader context of the TGD experience in the United States	**Course**: History of Psychology; Ethics & Professional Issues<br><br>**Content**: Psychology's role in problematic treatment of TGD individuals; current disparities and effects on TGD individuals' well-being<br><br>**LO**: Discuss the role of psychology in problematic understanding of gender diversity<br><br>Identify current disparities and effects on TGD individuals' well-being<br><br>**Reading:** Advancing the Practice of Pediatric Psychology with Transgender Youth: State of the Science, Ongoing Controversies, and Future Directions<br><br>**Activity:** Group discussion on psychology's role in problematic treatment of TGD individuals, current disparities, and effects on TGD individuals' well-being<br><br>Group discussion and/or written reflections on ways the field can improve experiences of TGD individuals	**Course:** Foundations of Multicultural Family Therapy<br><br>**Content:** Historical and present oppression of TGD youth, adults, and their families; minority stress, cisnormativity, and heteronormativity for creating health disparities; resilience and advocacy in TGD communities and families<br><br>**LO:** Describe relationships between intersectionality and minority stressors for LGBTQ+ communities.<br><br>**Reading:** Multiple Minority Stress: The Role of Proximal and Distal Stress on Mental Health Outcomes among Lesbian, Gay, and Bisexual People of Color<br><br>**Activity:** Role play with case examples for practicing identifying multiple minority stressors and cultural humility practices.	**Course**: Social Policy<br><br>**Content:** Social welfare policy in the United States; the income maintenance system and related programs in response to poverty, inequality, and racial and gender discrimination<br><br>**LO:** Apply behavioral skills to advocate for human rights and social, environmental, and economic justice.<br><br>Engage in practices that address disparities and advance human rights to promote social, racial, economic, and environmental justice<br><br>**Reading**: This is Social Work History<br><br>The Role Of The Transgender Community In The LGBT+ Rights Movement<br><br>**Activity**: Case study of a transgender client experiencing housing discrimination

	Physician Assistant	Nursing	Athletic Training	Nutrition and Dietetics	Clinical Psychology	Marriage & Family Therapy	Social Work
Legal, policy, and ethical implications for TGD people	**Course:** Professional and Psychosocial Issues of PA Practice; Ethics curriculum; Graduate seminar<br><br>**Content:** Overview of laws and typical insurance policies governing the care of transgender individuals, including transgender minors<br><br>**LO:** Describe laws and policies that affect the care of transgender individuals.<br><br>**Reading:** The Public Health Crisis State of Transgender Health Care and Policy<br><br>Health insurance coverage for gender-affirming care of transgender patients<br><br>**Activity: (Ethics curriculum):** Lecture and readings in *Professional and Psychosocial Issues* course; case based student led group discussion of ethical issues related to transgendered care in ethics curriculum; refresher lecture on insurance coverage, including for gender affirming care, in graduate seminar	**Course:** Health Care Policy<br><br>**Content:** Overview of policy, laws, and insurance coverage concerns including implications for minors<br><br>**LO:** Describe laws and policies that affect TGD persons including public life and access to health care.<br><br>**Reading:** The Public Health Crisis State of Transgender Health Care and Policy<br><br>**Activity:** Explore Movement Advancement Project and 2024 Anti-Trans Bills Tracker<br>Develop and/or rewrite an existing law or policy concerning TGD persons.	**Course:** Athletic Training Administration<br><br>**Content:** Policy development and implementation for athletic training practice<br><br>**LO:** Critically evaluate policies for inclusivity.<br><br>Develop policies and procedures for athletic training practice that are inclusive.<br><br>**Reading:**<br>Role of the AT - Part 1<br>Role of the AT - Part II<br><br>**Activity:** Evaluate examples of policies for inclusive language.<br><br>Design athletic training facilities with inclusive best practices.	**Course:** Community Nutrition<br><br>**Content:** Laws and policies regarding access to food assistance programs and the prevention of discrimination within those programs<br><br>**LO:** Address the barriers a TGD client may face when applying for Supplemental Nutrition Assistance Program (SNAP) Benefits.<br><br>**Reading:** Food Insecurity and Food Pantry use among Transgender and Gender Non-conforming People in the Southeast United States<br><br>**Activity:** Prepare an in-service for Missouri Department of Social Service employees that utilizes principles of gender-affirming communication.	**Course**: Ethics & Professional Issues; Human Diversity; Professional Development Seminar<br><br>**Content**: Legal restrictions in Missouri and surrounding states; how to navigate the legal system for best practice with TGD individuals; how to advocate for TGD clients<br><br>**LO**: Discuss current legal issues related to TGD individuals in Missouri<br><br>Identify at least one action item for addressing disparities for TGD individuals<br><br>**Reading:** Advancing the Practice of Pediatric Psychology with Transgender Youth: State of the Science, Ongoing Controversies, and Future Directions<br><br>**Activity:** Create case conceptualization of presenting concerns and legal and ethical considerations in working with TGD individuals (minors and adults); group discussion and/or written reflections on ways the field can assist with improving TGD policies	**Course:** Ethical & Legal Issues in Family Therapy<br><br>**Content:** Overview of unique legal and ethical issues for TGD youth and adults<br><br>**LO:** Identify unique barriers to healthcare and human rights due to local and national politics, policy, and laws.<br><br>**Reading:** LGBTQ Parent Families<br><br>The Public Health Crisis State of Transgender Health Care and Policy<br><br>**Activity:** Explore Movement Advancement Project and 2024 Anti-Trans Bills Tracker in small groups. Describe the ethical role and activities associated with advocacy as a mental health professional for TGD youth and adults.	**Course**: Policy Practice for Social Justice; Values and Ethics<br><br>**Content**: Tools and skills to change or enhance societal structures with oppressed and vulnerable groups<br><br>**LO**: Identify social policy at the local, state, and federal levels that impact well-being, service delivery, and access to social services for TGD communities.<br><br>Assess how social welfare and economic policies impact the delivery of and access to social services for TGD clients.<br><br>Apply critical thinking to analyze, formulate, and advocate for policies that advance human rights and social, economic, and environmental justice<br><br>**Reading**:<br>Advocating for Transgender Clients - National Association of Social Workers Ohio Chapter<br><br>**Activity**: Explore Movement Advancement Project and 2024 Anti-Trans Bills Tracker in small groups. Describe the ethical role and activities associated with advocacy as a social worker for TGD youth and adults.

	Physician Assistant	Nursing	Athletic Training	Nutrition and Dietetics	Clinical Psychology	Marriage & Family Therapy	Social Work
Counseling and communication skills with TGD clients	**Course:** Principles of the Medical Interview<br><br>**Content:** Communication skills for working with gender-diverse populations.<br><br>**LO:** Demonstrate best practice techniques to optimize care for transgender patients.<br><br>**Reading:** Communicating with transgender people in healthcare settings: clinical perspectives<br><br>**Activity:** Obtain a health history on a standardized patient who identifies as being transgender; as available, invite a transgender educator as a guest speaker,, or watch and discuss a first-person video of a TGD individual describing their experience	**Course:** Clinical Concepts; Health Promotion<br><br>**Content:** Clinical communication skills for gender-affirming care; clinician knowledge and attitudes<br><br>**LO:** Identify gender-affirming communication techniques for communicating with patients in the healthcare setting.<br><br>**Reading:** Affirmative Services for Transgender and Gender-Diverse People: Best Practices for Frontline Health Care Staff<br><br>**Activity:** Conduct a patient interview on a standardized client who is a transgender.	**Course:** Patient-Centered Care; Musculoskeletal Assessment & Management<br><br>**Content:** Clinical communication skills for patient-centered care, cultural competency, and inclusivity<br><br>**LO:** Demonstrate effective clinical communication skills that have respect for patient diversity.<br><br>**Reading:** Sensitive and Affirming Communication<br><br>**Activity:** Simulated/ standardized patient interviews with TGD patients	**Course:** Nutrition Counseling<br><br>**Content:** Best practices in gender-affirming communication<br><br>**LO**: Demonstrate effective gender-affirming communication skills when counseling a client.<br><br>**Reading:** Sensitive and Affirming Communication<br><br>**Activity:** Role play nutrition counseling with a peer; practice sharing name/pronouns and asking the patient to share theirs	**Course**: CVT; Clinical Interventions; Professional Development Seminar<br><br>**Content**: Best practices for working with TGD clients; special considerations in treatment; communication with non-affirming clients<br><br>**LO**: Identify at least 3 unique needs of TGD individuals in therapy.<br><br>Identify at least 3 considerations that may be needed for TGD individuals across different treatment modalities (e.g. CBT).<br><br>**Readings:** Guidelines for Psychological Practice With Transgender and Gender Nonconforming People<br><br>Affirmative Care of TGD Children and Adolescents<br><br>A Self-Assessment Tool for Cultivating Affirming Practices With Transgender and Gender-Nonconforming Clients, Supervisees, Students, and Colleagues<br><br>Assessment/Treatment Considerations for Working With LGBTQ+ Clients With PTSD<br><br>**Activity**: Create case conceptualization of presenting concerns and treatment considerations for TGD individuals; class discussion of readings; role playing	**Courses:** Introduction to Family Therapy; Group Counseling Theory and Practice<br><br>**Content:** Clinical communication skills for gender-affirming care; clinician knowledge and attitudes; working with families of TGD youth<br><br>**LO:** Demonstrate effective clinical communication skills that have respect for LGBTQ+ clients.<br><br>**Reading:** Clinical Guidelines for LGBTQIA-Affirming Marriage and Family Therapy<br><br>**Activity:** Watch recorded video of TGD youth with parents. Practice role play with TGD adults and youth.	**Courses:** Social Work Practice with Individuals, Families, and Groups; Advanced Clinical Practice with Individuals; Advanced Clinical Practice with Families and Groups<br><br>**Content**: Basics of gender-affirming communication, interviewing, and relationship building; professional use of self<br><br>**LO**: Explore the roles of race, ethnicity, culture, class, gender, sexual orientation, religion, physical or mental disability or illness, age, and national origin in social work practice.<br><br>Apply evidence-supported assessment and intervention approaches.<br><br>**Readings:** Clinical Social Work Practice Implications on the New Standards of Care by the World Professional Association of Transgender Health [WPATH]<br><br>Toolbox to Promote Healthy LGBT Youth<br><br>Affirmative Practice with Transgender Clients<br><br>**Activity**: Simulated/ standardized patient interviews with TGD clients

	Physician Assistant	Nursing	Athletic Training	Nutrition and Dietetics	Clinical Psychology	Marriage & Family Therapy	Social Work
Clinical assessment and TGD clients	**Course:** Graduate Seminar; Fundamentals of Genitourinary/Renal Medicine<br><br>**Content:** Physical examination skills in gender diverse individuals; clinical assessment of gender diverse individuals<br><br>**LO:** Describe effective techniques for communication and performing a physical exam in a gender diverse individual.<br><br>Complete a clinical assessment on a transgender individual who presents with an acute concern.<br><br>**Reading:** Transgender Patients and the Physical Examination<br><br>Standards of Care for Transgender and Gender Diverse People<br><br>**Activity:** As part of the summative evaluation required in the last months of the PA program, complete an assessment and develop a plan for an acute problem in a transgender individual.	**Course:** Health Assessment<br><br>**Content:** Best practices in assessment of the TGD patient<br><br>**LO:** Identify best practices for conducting a physical assessment with TGD patients.<br><br>**Reading:** Affirmative Services for Transgender and Gender-Diverse People: Best Practices for Frontline Health Care Staff<br><br>**Activity:** Conduct a physical assessment on a standardized client who is transgender.	**Course:** Musculoskeletal Assessment & Management<br><br>**Content:** Musculoskeletal assessment of injury<br><br>**LO:** Assess and diagnose musculoskeletal injury.<br><br>Perform a patient history interview with respect for patient diversity.<br><br>**Reading:** Transgender Patients and the Physical Examination<br><br>**Activity:** Simulated/ standardized patient cases that include gender diverse patients	**Course**: Nutrition Assessment<br><br>**Content**: Recommended method for collecting demographic data and name/pronouns in a nutrition assessment, best practices in conducting a nutrition focused physical exam.<br><br>**LO:** Demonstrate best practices in collecting sex, gender, sexual orientation, and pronouns during a standard nutrition assessment.<br><br>Demonstrate best practices in conducting a nutrition focused physical exam with a TGD patient.<br><br>**Reading:** Sensitive and Affirming Communication<br><br>Transgender Patients and the Physical Examination<br><br>**Activity:** Simulated/ standardized patient cases that include gender diverse patients	**Course**: Clinical Assessment I & II; CVT Teams<br><br>**Content**: Best practices for assessment with TGD individuals (with special attention to clinical interview, collection of developmental history, and scoring and interpreting gender norms)<br><br>**LO**: Articulate how gender influences cultural norms and how psychometric norms relate to TGD clients<br><br>**Reading:** Guidelines for Psychological Practice With Transgender and Gender Nonconforming People<br><br>Psychoeducational Assessment of Transgender and Gender Nonconforming (TGNC) Individuals: Recommendations for Best Practices<br><br>Which Box to Check: Assessment Norms for Gender and the Implications for Transgender and Nonbinary Populations<br><br>**Activity:** Group discussion/reflection of readings	**Course:** Diagnosis and Assessment in Family Therapy; Theory and Intervention in Human Sexuality<br><br>**Content:** Best practices in assessment and diagnosis of the TGD client<br><br>**LO:** Identify best practices for assessment and diagnosis with TGD persons.<br><br>**Reading:** The DSM-5 and the Politics of Diagnosing Transpeople<br><br>Standards of Care for the Health of Transgender and Gender Diverse People, Version 8<br><br>**Activity:** Review WPATH chapters 5, 6, and 7 on adult and child assessment through small and large group discussion; develop assessment protocol as a class activity.	**Course**: Diagnosis and Assessment<br><br>**Content**: Exploration, understanding, and critical analysis of the nature and diagnosis of psychopathology and the integration of the DSM-5-TR into clinical social work practice.<br><br>**LO**: Demonstrate competence in working with diverse populations and performing a bio-psycho-social-spiritual assessment that affirms population identity.<br><br>Demonstrate effective client assessment skills in clinical practice.<br><br>**Reading:** Psychiatry.org - History and Epidemiology.<br><br>How Gender Dysphoria and Incongruence Became Medical Diagnoses – A Historical Review<br><br>What the Past Suggests About When a Diagnostic Label Is Oppressive<br><br>**Activity:** Group discussion/reflection of readings

	Physician Assistant	Nursing	Athletic Training	Nutrition and Dietetics	Clinical Psychology	Marriage & Family Therapy	Social Work
Practicum and simulation with TGD patients/clients	**Course:** Clinical Elective<br><br>**Content**: Elective clerkship in a clinic focused on LGBTQ+ care<br><br>**LO:** Demonstrate a comprehensive understanding of the unique healthcare needs of gender diverse individuals.<br><br>**Reading:** Standards of Care for the Health of Transgender and Gender Diverse People, Version 8<br><br>**Activity:** Complete all aspects of the clinical elective, including obtaining patient histories, performing physical exams, creating assessments and care plans, and counseling patients.<br><br>Present a grand rounds style presentation to classmates and faculty of a transgender patient evaluated during the course of the clerkship.	**Course:** Final Practicum; Area of Concentration (AOC) Courses (e.g. Maternal/ Neonatal Health, Child Health, Psychiatric/ Mental Health,Older Adult, Adult)<br><br>**Content:** Best practices for working with TGD persons in nursing speciality areas<br><br>**LO:** Identify nursing implications for working with TGD clients in nursing specialty areas including Maternal/ Neonatal Health, Child Health, Public Health, Psychiatric/ Mental Health,Older Adult, and Adult Health Nursing.<br><br>Develop a plan of care for working with a TGD client who is admitted to the acute care setting for an acute health alteration.<br><br>**Reading:** Best practices for working with TGD clients in nursing speciality areas e.g. Providing Patient-Centered Care to the Transgender Community<br><br>**Activity:** Simulation with TGD client; Case studies in AOCs	**Course:** Clinical Practicum<br><br>**Content:** Clinical education experiences in athletic training<br><br>**LO:** Demonstrate appropriate supervised clinical care for diverse patient populations.<br><br>**Reading:**<br>Role of the AT - Part 1<br>Role of the AT - Part II<br><br>**Activity:** LGBTQ+ Safe Zone Training	**Course**: Medical Nutrition Therapy<br><br>**Content**: Nutrition-related implications of gender-affirming medical interventions (e.g. changes in body weight and composition)<br><br>**LO:** Complete the nutrition care process for a TGD patient.<br><br>**Reading:** The Impact of Gender-Affirming Hormone Therapy on Nutrition-Relevant Biomarkers<br><br>**Activity:** Nutrition care process for a TGD patient seen in the clinical setting	**Course**: Practicum; CVT<br><br>**Content**: Clinical and supervision experience with diverse populations; best practices for working with TGD individuals; special considerations in treatment; communication with non-affirming clients<br><br>**LO**: Demonstrate affirming psychological care for TGD clients<br><br>**Reading:** Guidelines for Psychological Practice With Transgender and Gender Nonconforming People<br><br>A Self-Assessment Tool for Cultivating Affirming Practices With Transgender and Gender-Nonconforming (TGNC) Clients, Supervisees, Students, and Colleagues<br><br>Assessment and Treatment Considerations for Working With LGBTQ+ Clients With PTSD<br><br>**Activity:** Utilize a supervisor rating form that assesses student competencies in working with TGD and diverse individuals; review Table 1 of "A Self-Assessment Tool" article with students and utilize as part of student feedback	**Courses:** Practicum and Internship<br><br>**Content:** Clinical and supervision experience with diverse populations<br><br>**LO:** Implement gender-affirming therapeutic approaches to support and empower TGD clients.<br><br>**Reading:** Clinical Guidelines for LGBTQIA-Affirming Marriage and Family Therapy<br><br>**Activity:** Take on at least 1 case at your practicum/ internship site or from the program's community clinic that is TGD; take part in group supervision.	**Courses:** Clinical Practicum I and II; Clinical Integrative Seminar<br><br>**Content:** Integration of theories, intervention models, and values and ethics with social work practice; peer sharing and debriefing on practice challenges<br><br>**LO:** Identify and apply relevant ethical principles in clinical practice.<br><br>Develop competence in ethical decision-making in clinical practice.<br><br>Engage in practices that address disparities and advance human rights to promote social, racial, gender, economic, and environmental justice.<br><br>Use practice experiences and theory to inform scientific inquiry and research.<br><br>**Reading:** Clinical Social Work Practice Implications on the New Standards of Care by the World Professional Association of Transgender Health [WPATH]<br><br>Toolbox to Promote Healthy LGBT Youth<br><br>Affirmative Practice with Transgender Clients<br><br>**Activity:** Internship that provides experience in working with TGD populations

	Physician Assistant	Nursing	Athletic Training	Nutrition and Dietetics	Clinical Psychology	Marriage & Family Therapy	Social Work
Research methods and SOGI data	**Course:** Evidence Based Medicine<br><br>**Content:** Recommended approach for collecting sex, gender, and sexual orientation data; a review of epidemiologic principles and concepts used to critically appraise an article related to transgender healthcare<br><br>**LO:** Describe the recommended approach for collecting sex, gender, and sexual orientation data.<br><br>Apply understanding of epidemiologic principles to critically appraise a journal article relating to transgender healthcare.<br><br>**Reading:** Sexual Orientation, Gender Identity, and Sex Development: Recommendations for Data Collection and Use in Clinical, Research, and Administrative Settings<br><br>**Activity:** Journal club article focused on transgender health	**Course:** Evidence Based Nursing<br><br>**Content:** Recommended approach for collecting sex, gender, and sexual orientation data<br><br>**LO:** Identify the gaps in research with regard to LGBTQ+ populations.<br><br>**Reading:** Sex and Gender in Health Research: Intersectionality Matters<br><br>**Activity:** Critically appraise a nursing research study and how the researchers captured demographic data; identify ways of collecting sex, gender, and sexual orientation data that more accurately capture the sample	**Course:** Clinical Research & Design<br><br>**Content:** Best practices for survey development, data collection, and reporting results<br><br>**LO:** Discuss best practices in survey/surveillance data collection for representation of diverse populations.<br><br>**Reading:** Measuring Sex, Gender Identity, and Sexual Orientation<br><br>**Activity:** Critically appraise literature including data collection methodologies and their representation of gender minority populations.	**Course**: Research Methods in Nutrition<br><br>**Content**: Recommended approach for collecting sex, gender, and sexual orientation data<br><br>**LO**: Evaluate how major nutrition surveillance programs in the U.S. are collecting sex, gender, and sexual orientation data.<br><br>**Reading**: Sex and Gender Data Collection in Nutrition Research<br><br>Measuring Sex, Gender Identity, and Sexual Orientation<br><br>**Activity**: Modify the demographic section of the National Health and Nutrition Examination Survey (NHANES) to utilize the recommended two-step method to collect SOGI data.	**Course**: Quantitative Research Methods; Qualitative Research Methods; Psychometric Theory; Research Vertical Teams (RVT)<br><br>**Content**: Recommended approach for collecting sex, gender, and sexual orientation data; considerations for impact of gender identity on hypotheses and results<br><br>**LO**: Identify at least 3 ways to appropriately include TGD identities in research (data collection, results, report writing, and future directions)<br><br>**Reading:** What Sexual and Gender Minority People Want Researchers to Know About Sexual Orientation and Gender Identity Questions: A Qualitative Study<br><br>A Self-Assessment Tool for Cultivating Affirming Practices With Transgender and Gender-Nonconforming (TGNC) Clients, Supervisees, Students, and Colleagues<br><br>**Activity**: Mock data set and write up – students use data to identify better ways to collect data, and practice interpreting and reporting data; utilize Table 1 of "A Self-Assessment Tool" article when designing research studies	**Course:** Research Method<br><br>**Content:** Recommended approaches for collecting and analysis of gender and sexual orientation data; ethics of research practice with marginalized communities; community engagement strategies; considerations for impact/intersection of gender identity on research hypotheses and results<br><br>**LO:** Critically evaluate research practices and ethics with marginalized communities.<br><br>**Reading/Resources:** Webinar: Collecting Sexual Orientation and Gender Identity Data<br><br>Guidance and Ethical Considerations for Undertaking Transgender Health Research and Institutional Review Boards Adjudicating this Research<br><br>**Activity:** Draft research proposal for a grant funder including 3 aims, methodology (inclusive of community engagement activities), and draft survey or interview protocol.	**Courses:** Evidence-Based Research and Program Evaluation<br><br>**Content:** Research literacy to engage in ethical and evidence-informed social work practice and evaluate programs through a social justice framework, with special attention to sex, gender, and sexual orientation data.<br><br>**LO:** Discuss best practices in survey/surveillance data collection for representation of diverse populations.<br><br>**Reading:** Measuring Sex, Gender Identity, and Sexual Orientation<br><br>Transgender Rights & Urgent Need for Social Work Advocacy<br><br>**Activity:** Critically appraise literature including data collection methodologies and their representation of gender minority populations

Notes: LO, learning outcome; TGD, transgender and gender diverse; SOGI, sex, sexual orientation, and gender identity
